# Single-Domain Antibody Probe with Low Renal Uptake for Claudin 18.2–Targeted PET Imaging of Gastric Cancer: Preclinical and Pilot Clinical Evaluations

**DOI:** 10.2967/jnumed.125.271704

**Published:** 2026-06

**Authors:** Meng Zheng, Haoqun Ma, Tao Xu, Huiwen Mu, Qingfeng Liu, Kaijie Zhang, Yicong Bian, Hua Zhang, Wei Li, Bin Zhang, Yuanyuan Shan, Xuanhui Peng, Songbing Qin, Yan Wang, Liyan Miao

**Affiliations:** 1 Department of Clinical Pharmacology, First Affiliated Hospital of Soochow University, Suzhou, China;; 2 National Institution of Drug Clinical Trial, First Affiliated Hospital of Soochow University, Suzhou, China;; 3 State Key Laboratory of Radiation Medicine and Protection, Soochow University, Suzhou, China;; 4 College of Pharmaceutical Sciences, Soochow University, Suzhou, China;; 5 Smart-Nuclide Biotech, Suzhou, China;; 6 Department of Oncology, First Affiliated Hospital of Soochow University, Suzhou, China;; 7 Department of Nuclear Medicine, First Affiliated Hospital of Soochow University, Suzhou, China; and; 8 Department of Radiation Oncology, First Affiliated Hospital of Soochow University, Suzhou, China

**Keywords:** CLDN18.2, single-domain antibody, targeted molecular imaging, gastrointestinal tumors, renal uptake

## Abstract

High renal uptake limits the clinical translation of Claudin 18.2 (CLDN18.2) nanobody probes. We aimed to develop and identify a single-domain antibody–based molecular probe to minimize nonspecific renal accumulation while maintaining high tumor affinity and effective uptake. **Methods:** A CLDN18.2-targeted nanobody, SNA014, was radiolabeled with ^68^Ga to yield [^68^Ga]Ga-SNA014. The binding capability of [^68^Ga]Ga-SNA014 was evaluated in vitro using flow cytometry, immunohistochemistry, and cell-binding assays. The biologic behavior of [^68^Ga]Ga-SNA014 in vivo was assessed through small-animal PET imaging, biodistribution studies, and blood pharmacokinetic analysis in human gastric adenocarcinoma xenograft models (both wild-type and CLDN18.2-overexpressing). Furthermore, a preliminary clinical evaluation of [^68^Ga]Ga-SNA014 was conducted in 3 patients with gastric cancer, including whole-body PET/CT imaging and radiation dosimetry analysis. **Results:** [^68^Ga]Ga-SNA014 was successfully synthesized with high radiochemical purity (>95%) and excellent stability both in vitro and in vivo. The probe demonstrated strong binding affinity and specificity toward AGS^CLDN18.2^ cells. Small-animal PET/CT images of AGS^CLDN18.2^ tumor–bearing mice exhibited high tumor and stomach uptake and low kidney uptake, and pretreatment with succinylated gelatin further reduced kidney retention. Blood clearance revealed a rapid elimination profile, with a half-life of 47.68 ± 1.83 min. In human PET/CT studies, distinct visualization of lesions was achieved up to 0.5 h postinjection. Dosimetry analysis revealed that the effective radiation dose of [^68^Ga]Ga-SNA014 was lower than that of standard [^18^F]FDG PET/CT. **Conclusion:** These findings demonstrated that [^68^Ga]Ga-SNA014 exhibits high affinity and specificity and excellent targeting performance and safety, enabling precise detection of CLDN18.2-overexpressing tumors.

## Visual Abstract

**Figure:**
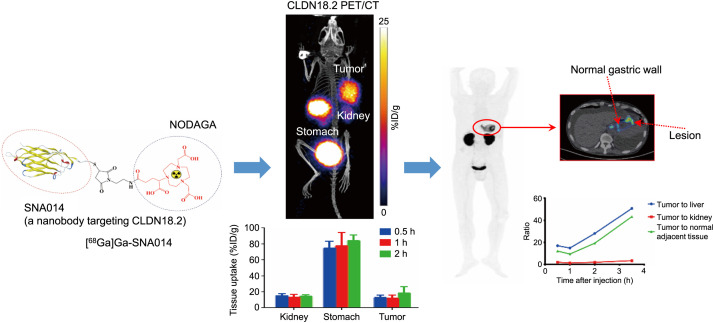

Gastric cancer ranks among the most prevalent malignant neoplasms worldwide, and its burden is particularly profound in China. Thus, the refinement and optimization of diagnostic and therapeutic strategies for gastric cancer carry significant clinical and public health importance (1). Recently, Claudin 18.2 (CLDN18.2) has become an extremely promising target in gastrointestinal malignancies (2,3). Several CLDN18.2-targeted drugs have been approved for clinical trials, such as the monoclonal antibody zolbetuximab (4) and antibody–drug conjugates like CMG901 and CAR-T (CT041) (5). However, clinical observations indicate that the therapeutic response to CLDN18.2-targeted treatment varies among patients and is positively associated with CLDN18.2 expression levels (6). The evaluation of CLDN18.2 expression is also crucial for guiding the design of personalized therapeutic strategies. Therefore, it is imperative to develop highly sensitive and specific methods for the detection of CLDN18.2.

In contrast to conventional detection techniques, such as immunohistochemistry, molecular imaging leverages radiolabeled probes to noninvasively delineate the spatial pattern of CLDN18.2 expression, thus enabling superior patient stratification, dynamic treatment monitoring, and precision-guided therapy. Single-domain antibodies, also known as nanobodies—the smallest functional antibody fragments derived from the variable heavy-chain domains of camelid antibodies—exhibit distinct advantages, such as low molecular weight, superior tissue permeability, strong antigen-binding affinity, and rapid systemic elimination (7). These features render them ideal for labeling with short-lived radionuclides (e.g., ^68^Ga, ^18^F), supporting fast, high-resolution molecular imaging (8). Representative CLDN18.2-targeted PET tracers—including [^68^Ga]Ga-NC-BCH (9), [^68^Ga]Ga-PMD22 (10), and the hu19V3 series ([^68^Ga]Ga-NOTA-hu19V3, [^64^Cu]Cu-NOTA-hu19V3, and [^18^F]F-hu19V3) (11)—have shown excellent agreement between their in vivo biodistribution and histopathologic validation in preclinical models.

Despite their favorable pharmacokinetic profiles, radiolabeled nanobody probes face a major challenge related to renal handling (12–14). Their hydrophilic nature and low molecular weight lead to nearly complete glomerular filtration, followed by partial reabsorption in proximal tubular cells. Consequently, the kidneys are exposed to disproportionately high doses of radiation, resulting in potential nephrotoxicity and long-term renal injury, ultimately limiting the administrable activities of therapeutic radionuclides. Such dose-limiting renal toxicity remains a key obstacle to the broader clinical translation of radiolabeled nanobody-based therapeutics.

To address this challenge, we developed and identified a single-domain antibody–based molecular probe, ^68^Ga-labeled nanobody ([^68^Ga]Ga-SNA014), which markedly reduces renal uptake while preserving robust tumor-targeting capability. This study aimed to evaluate the ability of [^68^Ga]Ga-SNA014 to characterize CLDN18.2 expression and investigate its pharmacokinetic behavior in both preclinical and clinical settings. By mitigating dose-dependent nephrotoxicity, this approach holds the potential to accelerate clinical translation, offering a promising strategy to enhance the safety profile of CLDN18.2-targeted radiopharmaceuticals in future therapeutic applications.

## MATERIALS AND METHODS

Detailed descriptions of the materials and experimental procedures are provided in the supplemental materials, available at http://jnm.snmjournals.org.

All applicable international, national, or institutional guidelines for the care and use of animals were followed. All animal procedures were conducted under a protocol approved by the Soochow University Institutional Animal Care. All procedures involving human participants complied with institutional and national ethical standards and adhered to the principles of the Declaration of Helsinki. The study protocol was approved by the Research Ethics Board of the First Affiliated Hospital of Soochow University (July 10, 2024; no. 255), and written informed consent was provided by all participants before enrollment (ClinicalTrials.gov identifier NCT06646783).

### PET/CT Imaging of [^68^Ga]Ga-SNA014 in Tumor-Bearing Mice

Tumor-bearing mice were divided into 3 groups (8 per group): CLDN18.2-overexpressing xenografts (AGS^CLDN18.2^), wild-type AGS xenografts (AGS^WT^), and 1000-fold excess AGS^CLDN18.2^ blocking. All animals received an intravenous injection of 100 μL of [^68^Ga]Ga-SNA014 (15 μg/200 μCi) via the tail vein. For the blocking group, AGS^CLDN18.2^ tumor–bearing mice were coinjected with 1000-fold mass excess of unlabeled SNA014. At 0.5, 1.5, and 4 h postinjection, mice were anesthetized with isoflurane (3% induction, 1.5% maintenance) and underwent PET/CT scanning. PET images were reconstructed using Avatar 3 software (Pingseng Healthcare) with ordered-subset expectation maximization algorithm.

### Patient Study of [^68^Ga]Ga-SNA014

In total, 3 male patients (P101, P102, and P103) with gastric cancer were enrolled in this study (mean age, 61.33 ± 9.46 y) (Supplemental Table 1). The primary gastric lesions had been surgically resected in all 3 patients, among whom patient P103 had undergone total gastrectomy. All patients received intravenous injections of [^68^Ga]Ga-SNA014. Specifically, P101 received 0.8 mg (103.97 MBq), P102 received 1.5 mg (176. 34 MBq), and P103 received 1.5 mg (127.72 MBq). To evaluate the in vivo uptake kinetics of [^68^Ga]Ga-SNA014 and the temporal stability of lesion-to-background contrast, each patient underwent 4 whole-body PET/CT scans at 0.5, 1, 2, and 3.5–4 h postinjection.

### Statistical Analysis

All data were analyzed using Prism 9.0 (GraphPad Software). Data are presented as the mean ± SD. A *P* value of less than 0.05 was considered statistically significant.

## RESULTS

### Synthesis and Characterization of SNA014

As depicted in Supplemental Figure 1A, SNA014 demonstrates specific binding to CLDN18.2 on the AGS^CLDN18.2^ cell line, with superior binding affinity compared with IMAB362, a commercially available monoclonal antibody targeting CLDN18.2. Notably, it does not bind to the AGS^WT^ cell line. To further validate the binding specificity of SNA014 to the CLDN family, particularly CLDN18.2 and Claudin 18.1 (CLDN18.1), different concentrations of SNA014 were incubated with 293T^CLDN18.1^ and 293T^CLDN18.2^ cells. Flow cytometry results revealed that SNA014 specifically binds to CLDN18.2 on the 293T^CLDN18.2^ cell line, with a half-maximal effective concentration of 5.55 nmoL/L, while showing no binding to 293T^CLDN18.1^ cells (Supplemental Fig. 1B). This suggests that SNA014 does not interact with CLDN18.1 but rather binds exclusively to CLDN18.2. Additionally, SNA014 exhibited strong cross-reactivity with CLDN18.2 in gastric tissues across various animals, such as rats, rabbits, beagles, guinea pigs, humans, and mice (Supplemental Fig. 1C).

### Molecular Characteristics of Conjugation

SNA014 is a single-domain antibody, with an approximate molecular weight of 14 kDa, which was further determined to be 13,451.488 Da by liquid chromatography–mass spectrometry (Supplemental Fig. 2A). NODAGA-SNA014, chelated with a double-maleimide-NODAGA chelator, exhibited a molecular weight of 13,829.704 Da (Supplemental Fig. 2B). Size-exclusion–high-performance liquid chromatography analysis revealed that the retention times of SNA014 and NODAGA-SNA014 were nearly identical, with no additional peaks detected (Supplemental Fig. 2C). This suggests that SNA014 was successfully conjugated to maleimide-NODAGA. Enzyme-linked immunosorbent assay results showed that the half-maximal effective concentration values for NODAGA-SNA014 binding to CLDN18.2 were not significantly different from those of SNA014 (9.84 nmoL/L vs. 5.33 nmoL/L; Supplemental Fig. 2D). These results further indicated that both SNA014 and NODAGA-SNA014 exhibit strong binding to CLDN18.2 and that the presence of the maleimide-NODAGA did not affect the affinity of SNA014 for CLDN18.2.

### Radiosynthesis and Characterization of [^68^Ga]Ga-SNA014

**FIGURE 1.**
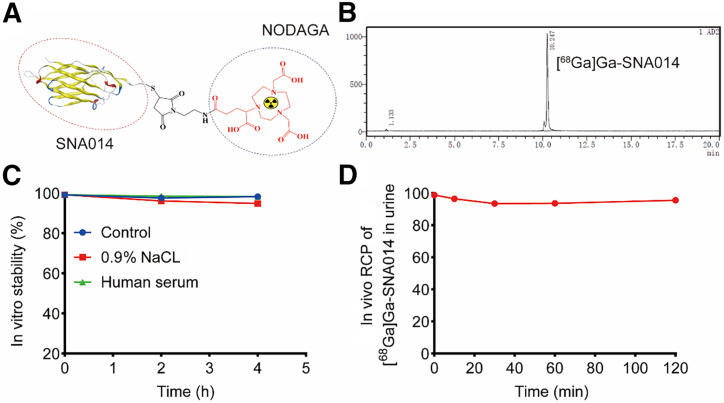
Quality control and in vitro stability of [^68^Ga]Ga-SNA014. (A) Illustration of SNA014 conjugation and radiolabeling with ^68^Ga. (B) Radiolabeling efficiency of [^68^Ga]Ga-SNA014. (C) In vitro stability of [^68^Ga]Ga-SNA014. (D) In vivo stability of [^68^Ga]Ga-SNA014 in mice urine.

[^68^Ga]Ga-SNA014 was synthesized through a synthetic method, with a radiochemical yield of 95.00% ± 0.02% (Figs. 1A and 1B). In vitro stability studies revealed that the radiochemical purity of [^68^Ga]Ga-SNA014 remained above 99% after incubation for 0, 2, and 4 h at room temperature in both 0.9% NaCl solution (pH 7.4) and human serum (Fig. 1C). No significant differences were observed compared with the initial [^68^Ga]Ga-SNA014. After administration of [^68^Ga]Ga-SNA014 in normal mice, radiochemical purity in urine remained above 90% at 0 min, 10 min, 30 min, 1 h, and 2 h postinjection (Fig. 1D). The excellent in vivo and in vitro stabilities suggest that the structural modifications and labeling method for SNA014 are feasible.

### Cell Uptake and Competitive Binding Assay

**FIGURE 2.**
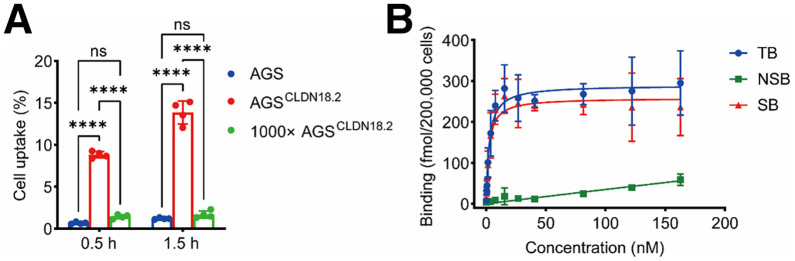
(A) Cellular uptake of [^68^Ga]Ga-SNA014 in AGS^WT^ and AGS^CLDN18.2^ cells. (B) Saturation binding analysis of [^68^Ga]Ga-SNA014 probe in AGS^CLDN18.2^ cells. ns = not significant; NSB = nonspecific binding; SB = specific binding; TB = total binding. ****P < 0.005.

To validate the specificity and affinity of [^68^Ga]Ga-SNA014, cell uptake and saturation binding experiments were conducted in AGS^CLDN18.2^ and AGS^WT^ cells. After 1.5 h of incubation, the mean uptake of [^68^Ga]Ga-SNA014 in AGS^CLDN18.2^ cells was 13.85% ± 1.38%, significantly higher than the mean uptake observed at 0.5 h, which was 8.82% ± 0.38% (*P* < 0.005) (Fig. 2A). Regardless of the incubation time, the uptake of [^68^Ga]Ga-SNA014 in AGS^WT^ cells remained low, with mean values of 0.67% ± 0.12% at 0.5 h and 1.20% ± 0.10% at 1.5 h. Notably, compared with the unblocked group, the uptake of [^68^Ga]Ga-SNA014 in AGS^CLDN18.2^ cells was significantly reduced after blocking with a 1000-fold excess of unlabeled SNA014 (1.68% ± 0.43% at 1.5 h, *P* < 0.001).

In addition, saturation binding experiments revealed that the probe’s maximal binding capacity and equilibrium dissociation constant were 258.60 fmol/200,000 cells and 2.18 nM, respectively (Fig. 2B). These findings confirm that [^68^Ga]Ga-SNA014 exhibits high specificity and strong affinity for the CLDN18.2 protein.

### Small-Animal PET/CT, Biodistribution, and Pharmacokinetics

**FIGURE 3.**
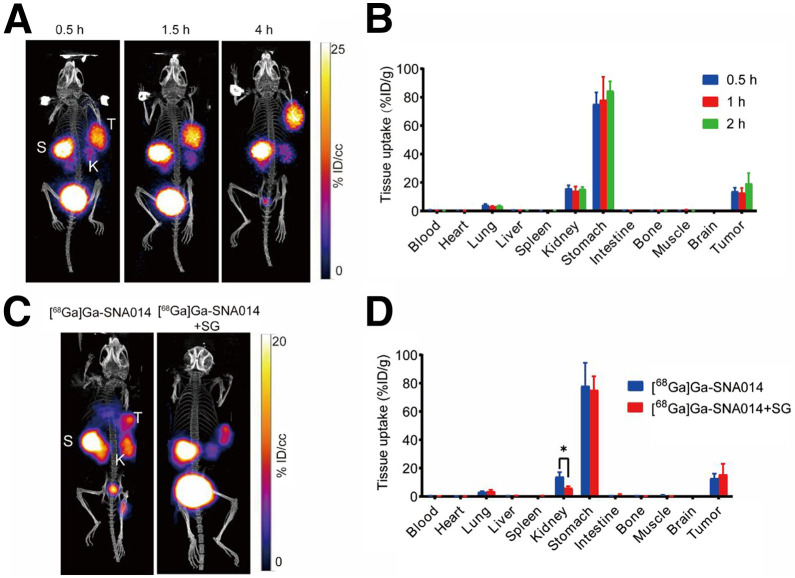
Small-animal PET imaging of AGS^CLDN18.2^ tumor–bearing mice after administration of [^68^Ga]Ga-SNA014. (A) Representative PET images acquired at 0.5, 1.5, and 4 h postinjection in AGS^CLDN18.2^ tumor–bearing mice. (B) Ex vivo biodistribution of [^68^Ga]Ga-SNA014 in AGS^CLDN18.2^ tumor–bearing mice at 0.5, 1, and 2 h postinjection. (C) Small-animal PET imaging of [^68^Ga]Ga-SNA014 in AGS^CLDN18.2^ tumor–bearing mice 1 h postinjection, with or without administration of SG. (D) Ex vivo biodistribution of [^68^Ga]Ga-SNA014 in AGS^CLDN18.2^ tumor–bearing mice 1 h postinjection, with or without administration of SG (*n* = 3). K = kidney; S = stomach; T = tumor.

To evaluate the ability of the tracer to visualize tumor CLDN18.2 expression, PET imaging of AGS^CLDN18.2^ and AGS^WT^ tumor–bearing mice was performed with [^68^Ga]Ga-SNA014 at 30 min, 1.5 h, and 4 h. As shown in Figure 3A, AGS^CLDN18.2^ tumor–bearing mice exhibited high tumor and stomach uptake and low kidney uptake. In contrast, markedly reduced uptake of [^68^Ga]Ga-SNA014 at the tumor site was observed in both the AGS^WT^ group and the AGS^CLDN18.2^ blocking group (Supplemental Fig. 3). Ex vivo biodistribution studies showed that [^68^Ga]Ga-SNA014 exhibited high radioactivity accumulation in CLDN18.2-positive tumors. Mean tumor uptake was relatively high (13.92 ± 2.44 %ID/g at 0.5 h, 12.95 ± 3.20 %ID/g at 1 h, and 19.41 ± 7.24 %ID/g at 2 h), whereas kidney uptake of [^68^Ga]Ga-SNA014 was comparable (∼15 %ID/g) at 0.5, 1, and 2 h (Fig. 3B).

It has been reported that succinylated gelatin (SG) and lysine can reduce renal uptake of radiotracers (15). Our preliminary studies demonstrated that l-lysine did not reduce the renal uptake of [^68^Ga]Ga-SNA014 (Supplemental Fig. 4). Thus, we investigated the potential role of SG in regulating renal uptake. The results showed that administration of SG significantly reduced the renal uptake of [^68^Ga]Ga-SNA014 in AGS^CLDN18.2^ tumor–bearing mice at 1 h postinjection (Fig. 3C). Ex vivo biodistribution analysis revealed that SG could significantly reduce renal uptake by 56.2% without affecting tumor uptake (*P* < 0.05), with no observable significant impact on uptake in other tissues, such as the tumor or stomach (Fig. 3D). This phenomenon was also confirmed in non–tumor-bearing mice (Supplemental Figs. 5 and 6). This result suggests that SG may play a role in reducing the renal uptake of [^68^Ga]Ga-SNA014.

As illustrated in Supplemental Fig. 7, [^68^Ga]Ga-SNA014 followed a biphasic pharmacokinetic profile in both mouse models. In AGS^CLDN18.2^ mice, the mean half-life and area under the curve (AUC) were 47.68 ± 1.83 min and 422.30 ± 49.39 min·%ID/g, respectively.

### PET/CT Imaging in Patients with Gastric Cancer

Three patients with gastric cancer (P101, P102, and P103) underwent whole-body PET/CT imaging after intravenous administration of [^68^Ga]Ga-SNA014. Patient characteristics are summarized in Supplemental Table 1. No tracer-related adverse events were observed after the administration of [^68^Ga]Ga-SNA014.

**FIGURE 4.**
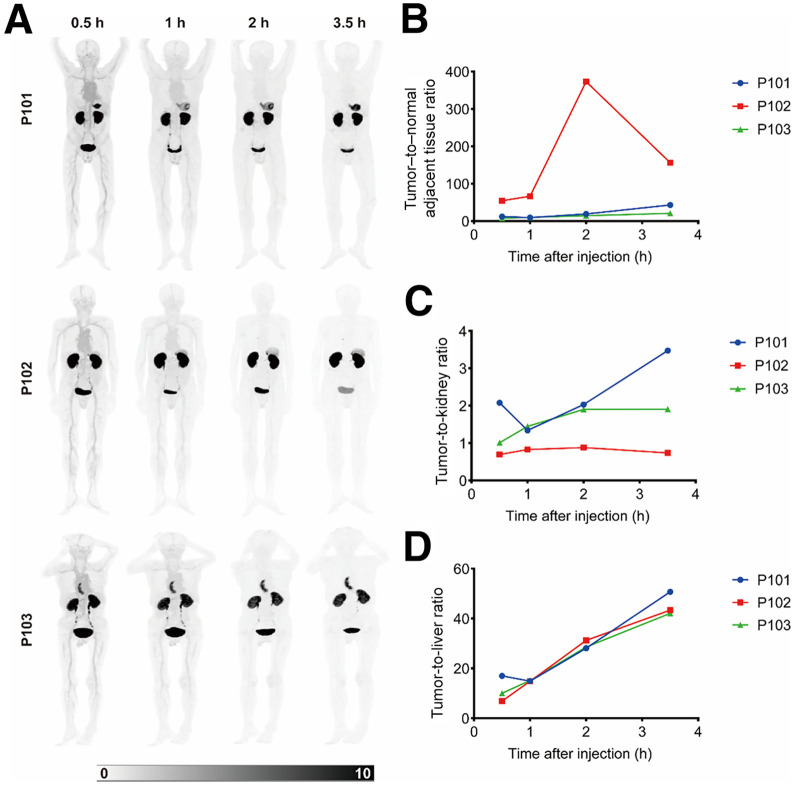
(A) Maximum-intensity-projection PET/CT images of 3 patients with gastric cancer (P101, P102, and P103) after administration of [^68^Ga]Ga-SNA014, acquired using 2-m long–axial-field-of-view PET/CT system. (B) Time curve of tumor–to–normal adjacent tissue ratio. (C) Time curve of tumor-to-kidney ratio. (D) Time curve of tumor-to-liver ratio.

[^68^Ga]Ga-SNA014 exhibited substantially higher accumulation in lesions than in adjacent normal tissues (Fig. 4; Supplemental Figs. 8 and 9; Supplemental Table 2). Within 3.5 h after injection, the SUV_mean_ in the kidneys of all 3 patients remained below 15, and those in the liver were less than 1.5. Notably, from 1 h onward, tumor uptake in most patients remained relatively stable, whereas radiotracer activity in background tissues, including the liver, progressively declined, leading to a marked enhancement in tumor-to-background contrast. Furthermore, with the exception of patient P101, patients P102 and P103 demonstrated an initial increase in the tumor-to-kidney ratio, followed by stabilization after 2 h. The tumor–to–peritumoral tissue ratio increased gradually in patients P101 and P103, whereas in patient P102 it reached a peak value of 373.33 at 2 h and then decreased. Collectively, these results indicate that [^68^Ga]Ga-SNA014 imaging at 1–2 h postinjection may afford diagnostically valuable tumor contrast. Its low renal retention coupled with pronounced tumor selectivity further highlights a favorable biodistribution profile for clinical applications, reinforcing its translational potential in gastric cancer molecular imaging.

### Dosimetry of Normal Tissue

The highest organ doses were estimated in the kidneys, stomach, and liver. Effective doses for P101, P102, and P103 were 2.97, 3.42, and 1.79 mSv, respectively (Table 1), all notably lower than the typical radiation exposure of conventional [^18^F]FDG PET/CT (7.0–14.0 mSv) (16). These findings indicate that whole-body PET imaging not only reduces overall radiation burden but also supports the translational development of this radiopharmaceutical.

**TABLE 1.** Estimated Human Organ Absorbed Radiation Dosimetry of [^68^Ga]Ga-SNA014

	[^68^Ga]Ga-SNA014 Dose (mSv/MBq)		
Variable	Patient P101	Patient P102	Patient P103
Effective dose	0.0286	0.0194	0.014
Tissue			
Liver	0.0082	0.0078	0.007
Bone marrow	0.0113	0.007	0.0072
Kidney	0.131	0.221	0.0818
Stomach wall	0.0273	0.0142	0.0063
Whole body	0.0099	0.0071	0.0066

## DISCUSSION

In this study, we investigated the use of SNA014, derived from alpaca immunization and screened by phage display, which exhibits high binding affinity to CLDN18.2 rather than CLDN18.1. [^68^Ga]Ga-SNA014 can be synthesized conveniently with a high radiochemical yield, excellent radiochemical purity, and moderate molar activity, while demonstrating good in vitro and in vivo stability. In vitro cell uptake and binding studies indicated that [^68^Ga]Ga-SNA014 exhibits strong affinity for CLDN18.2 and can specifically bind to CLDN18.2-positive cells, with an equilibrium dissociation constant of 2.18 nM and a maximal binding capacity of 258.60 fmol/200,000 cells. These promising results have prompted us to further investigate the potential of [^68^Ga]Ga-SNA014 for PET imaging for the evaluation of CLDN18.2 expression.

PET/CT imaging with [^68^Ga]Ga-SNA014 revealed prominent accumulation in renal, urinary bladder, and nonpathological gastric mucosa. Tracer uptake was markedly reduced across most tissues in the presence of a 1000-fold excess blocking dose, particularly within AGS^CLDN18.2^-positive tumors. This finding demonstrates the high specificity of [^68^Ga]Ga-SNA014 for CLDN18.2. In contrast, minimal tumor uptake was observed in AGS^WT^ xenografts, confirming that [^68^Ga]Ga-SNA014 selectively targets CLDN18.2-expressing lesions. [^68^Ga]Ga-SNA014 exhibited pronounced accumulation in the gastric mucosa, attributed to the small molecular size of nanobodies, which allows them to efficiently penetrate the gastric mucosal barrier and access the densely packed and otherwise concealed CLDN18.2 epitopes. This pattern was highly consistent with that observed for [^68^Ga]Ga-NC-BCH and [^68^Ga]Ga-PMD22 (9,10).

Moreover, the markedly lower uptake of [^68^Ga]Ga-SNA014 in the liver, spleen, and blood compared with other CLDN18.2-targeted antibody probes ([^89^Zr]Zr-DFO-TST001 (17) and [^124^I]I-18B10 (10 L) (18)) further supports the influence of molecular size on tracer biodistribution. Therefore, nanobody-based tracers, such as [^68^Ga]Ga-SNA014, exhibit reduced nonspecific retention and more favorable pharmacokinetics (19,20). Subsequently, we present, to our knowledge, the first reported clinical evaluation of [^68^Ga]Ga-SNA014 PET/CT in human subjects. Consistent with preclinical findings, [^68^Ga]Ga-SNA014 exhibited predominant accumulation in lesions, nonneoplastic gastric tissue, kidneys, and urinary bladder in patients P101 and P102. Whole-body PET/CT imaging revealed significantly higher tracer uptake in gastric tumor tissue compared with adjacent nonneoplastic mucosa (e.g., patient P101 demonstrated ∼43.25-fold greater uptake in lesions vs. paraneoplastic tissue at 3.5 h postinjection), aligning with established CLDN18.2 expression patterns. This differential uptake profile supports the diagnostic utility of SNA014 and suggests therapeutic potential. Crucially, in patient P103 with prior total gastrectomy, [^68^Ga]Ga-SNA014 specifically localized to metastatic lesions, validating its capacity as a clinical precursor for CLDN18.2-targeted therapies.

Additionally, [^68^Ga]Ga-SNA014 exhibited a certain degree of nonspecific accumulation in multiple organs, particularly the kidneys, consistent with the typical biodistribution pattern of nanobody-based tracers undergoing rapid renal clearance via the kidney–urine pathway (12,13,21). Ex vivo biodistribution results in preclinical studies showed that renal uptake of [^68^Ga]Ga-SNA014 remained relatively stable at 0.5, 1, and 2 h postinjection, with an average of approximately 15 %ID/g, which is significantly lower than that reported for other CLDN18.2-targeting radiolabeled nanobodies, such as [^68^Ga]Ga-NC-BCH (207.66 ± 19.99 %ID/g at 2 h), [^68^Ga]Ga-PMD22 (372.09 ± 24.46 %ID/g at 2 h), and [^68^Ga]Ga-NOTA-hu19V3 (30.0 ± 2.81 %ID/g at 1 h). This advantage has also been confirmed in clinical studies, with an SUV_mean_ of [^68^Ga]Ga-SNA014 of approximately 10–15 in the kidneys, markedly lower than that of [^68^Ga]Ga-NC-BCH (SUV_mean_, >60) (9) and [^68^Ga]Ga-PMD22 (SUV_mean_, 73.40) (10) and lower than that of the commonly used tracer [^68^Ga]Ga-PSMA-617 (SUV_mean_, 15.60) (22).

Nevertheless, residual radioactivity in the kidneys may result in a relatively high absorbed dose, potentially limiting the clinical application of radiopharmaceuticals. Notably, in the AGS^CLDN18.2^ xenograft model, coadministration of SG reduced renal uptake by 56.2% without affecting tracer accumulation in the tumor at 1 h postinjection, suggesting that this strategy could effectively lower renal radiation exposure without compromising imaging efficacy. On the basis of dosimetric simulations, administration of a single 74-MBq dose of [^68^Ga]Ga-SNA014 in men corresponds to an effective whole-body dose of approximately 1.53 mSv and a renal equivalent dose of 10.7 mSv, which was markedly lower than the renal equivalent dose with [^68^Ga]Ga-PMD22 (61.05 mSv) (10), [^68^Ga]Ga-NC-BCH (121.36 mSv) (9), and [^68^Ga]Ga-PSMA-617 (15.2 mSv) (22), with all critical organ doses remaining below the human research limits (30 mSv) specified by the Food and Drug Administration under 21 Code of Federal Regulations 361.1. Collectively, [^68^Ga]Ga-SNA014 demonstrates a relatively low renal radiation burden and favorable dosimetric safety while maintaining high tumor uptake in the stomach, providing a solid foundation for the further development of therapeutic radiolabeled derivatives (e.g., [^177^Lu]Lu-SNA014) and their clinical translation in CLDN18.2-targeted therapies. These results indicate that [^68^Ga]Ga-SNA014 PET/CT imaging is safe and noninvasive and can accurately detect tumors with CLDN18.2 overexpression, making it suitable for imaging analyses of gastrointestinal tumors.

However, several challenges remain. Although [^68^Ga]Ga-SNA014 successfully delineates tumors, its small molecular size, which facilitates efficient tumor penetration, also leads to notable uptake in normal gastric mucosa and the kidneys. This dual distribution underscores the difficulty in achieving precise diagnostic specificity and highlights potential therapeutic challenges. Therefore, reducing the physiologic uptake of [^68^Ga]Ga-SNA014 in the gastric mucosa and kidneys is of great significance for the future development of therapeutic radiolabeled nanobody drugs to minimize radiation-induced toxicity to these organs. In this regard, our preclinical dose-escalation studies demonstrated that optimization of molar activity (i.e., adjustment of the amount of compound in the administered PET probe solution) may represent an alternative strategy to increase the tumor–to–normal stomach uptake ratio (Supplemental Fig. 10). These findings indicate that dose-optimization studies in clinical trials are warranted to determine the optimal predosing strategy to overcome excessive uptake in normal gastric tissues. Furthermore, since tumor biopsy samples for assessing CLDN18.2 expression were not collected during imaging or treatment, further investigation is needed to clarify the correlation between tumor uptake and CLDN18.2 expression levels in future studies. This would help confirm the feasibility of using [^68^Ga]Ga-SNA014 for PET imaging of CLDN18.2 and ultimately guide CLDN18.2-targeted therapies.

## CONCLUSION

We developed a CLDN18.2-targeted nanobody probe for precise molecular imaging of gastrointestinal tumors. This probe has a high tumor affinity and specificity with low renal uptake, enabling clear visualization of CLDN18.2-positive lesions with reduced background signals in preclinical and clinical settings. The design showcases a new strategy for optimizing nanobody-based tracers toward safer and more accurate clinical translation.

## DISCLOSURE

This study was supported by the Regional Innovation and Development United Fund Project of the National Natural Science Foundation of China (U24A20765, T232100), Noncommunicable Chronic Diseases-National Science and Technology Major Project (2024ZD0525900), Jiangsu Provincial Science and Technology Plan Special Fund (BM2023003), Jiangsu Provincial Medical Key Discipline Program (ZDXK202247), Priority Academic Program Development of the Jiangsu Higher Education Institutes, Suzhou Basic Research Special Project (KJXW2023004, SSD2024039, SYWD2024246, SYWD2025233), Suzhou Gusu Health Talent Research Project (GSWS2022013), and Project of State Key Laboratory of Radiation Medicine and Protection at Soochow University (GZK12025052). No other potential conflict of interest relevant to this article was reported.

## Notes

KEY POINTS **QUESTION:** Can a CLDN18.2-specific nanobody probe be developed that preserves effective tumor uptake while significantly reducing renal accumulation, thereby maintaining imaging performance and improving clinical translatability? **PERTINENT FINDINGS:** We developed a CLDN18.2-targeted nanobody probe for precise molecular imaging of gastrointestinal tumors. [^68^Ga]Ga-SNA014 specifically binds CLDN18.2 and shows high tumor uptake (up to 19.41%ID/g) and lower renal uptake (∼15%ID/g) in preclinical models. Clinically, it visualizes lesions with high specificity (SUV_max_, 20.76) and low radiation dose, outperforming existing CLDN18.2 tracers. **IMPLICATIONS FOR PATIENT CARE:** [^68^Ga]Ga-SNA014 achieves high tumor uptake while maintaining exceptionally low renal accumulation, thereby reducing nephrotoxicity and radiation exposure. These advantages enable more accurate assessment of tumor burden and therapeutic response and may facilitate earlier detection of CLDN18.2-positive disease.
